# Inflammation-related genes *S100s*, *RNASE3*, and *CYBB* and risk of leukemic transformation in patients with myelodysplastic syndrome with myelofibrosis

**DOI:** 10.1186/s40364-021-00304-w

**Published:** 2021-07-02

**Authors:** Minghua Hong, Junqing Wu, Lifeng Ma, Xiaoping Han, Ting Lu, Zhaoming Wang, Jing Zhao, Lizhen Liu, Huarui Fu, Weijia Huang, Weiyan Zheng, Jingsong He, Guoqing Wei, Huanping Wang, Zhimei Chen, He Huang, Zhen Cai, Guoji Guo, Jie Sun

**Affiliations:** 1 Bone Marrow Transplantation Center, the First Affiliated Hospital, Zhejiang University School of Medicine; Institute of Hematology, Zhejiang University; Zhejiang Province Engineering Laboratory for Stem Cell and Immunity Therapy; Liangzhu Laboratory, Zhejiang University Medical Center, 1369 West Wenyi Road, 310003 Hangzhou, China; 2 Center for Stem Cell and Regenerative Medicine, Stem Cell Institute, School of Medicine, Zhejiang University, 310058 Hangzhou, China; 3 Pathology Department, the First Affiliated Hospital, School of Medicine, Zhejiang University, 310003 Hangzhou, China

**Keywords:** Myelodysplastic syndrome, Myelofibrosis, Leukemic transformation, Single-cell sequence, Inflammation

## Abstract

Myelodysplastic syndrome with myelofibrosis (MDS-MF) has been associated with an inferior prognosis compared with MDS without MF. However, MDS-MF is not listed independently as a subtype of MDS, and its clinical and genetic characteristics remain poorly understood. We retrospectively compared 53 patients with MDS-MF (44 MF grade 1/MF_1_; 9 MF grade 2–3/MF_2 − 3_) and 31 with *de novo* MDS without MF (MDS). The leukemic transformation risks of both MDS-MF_2 − 3_ and MDS-MF_1_ were increased compared with the MDS group. To identify the potential mechanisms responsible for the leukemic transformation of MDS-MF, we performed single-cell sequencing for one MDS-MF_2 − 3_ patient before and after leukemic transformation to explore the variations in gene expression levels. In addition to upgraded expression levels of acute myeloid leukemia-related genes during leukemic transformation, expression levels of some inflammation-related genes (such as *S100s*, *RNASE3*, and *CYBB*) were also increased, and inflammation-related pathways were up-regulated. These results suggest that inflammation-related genes and pathways may play an important role in the leukemic transformation of MDS-MF.

**Supplementary Information:**

The online version contains supplementary material available at 10.1186/s40364-021-00304-w.

To the Editor

Myelodysplastic syndrome (MDS) with myelofibrosis (MDS-MF) accounts for up to 50 % of cases of MDS [1], and differs from MDS without MF in terms of clinical performance, treatment tolerance, and survival [2]. MDS with severe MF (MF grade = 2–3) is considered as an independent risk factor for *de novo* MDS [3]; however, MDS-MF is not independently listed as a subtype of MDS according to the 2016 World Health Organization classification, indicating the need for further investigation.

We retrospectively enrolled 53 patients with MDS-MF (44 MF grade 1/MF_1_; 9 MF_2 − 3_) and 31 patients with *de novo* MDS without MF (MDS). There were no significant differences among the MDS, MDS-MF_1_, and MDS-MF_2 − 3_ groups in terms of age, sex, MDS subtypes, IPSS risk levels, and treatment strategies, except for a higher rate of poor karyotypes in the MDS-MF_2 − 3_ compared with the MDS and MDS-MF_1_ groups (with no difference between the MDS and MDS-MF_1_ groups) (Supplementary Table 1). Not only MDS-MF_2 − 3_, but also MDS-MF_1_ at IPSS low/int-1 risk, had a shorter leukemic transformation time compared to the MDS group (Fig. 1), suggesting that patients with low/int-1 MDS with even mild MF required chemotherapy to avoid disease progression. Detailed patients’ clinical data are shown in Supplementary file 1.

**Fig. 1:**
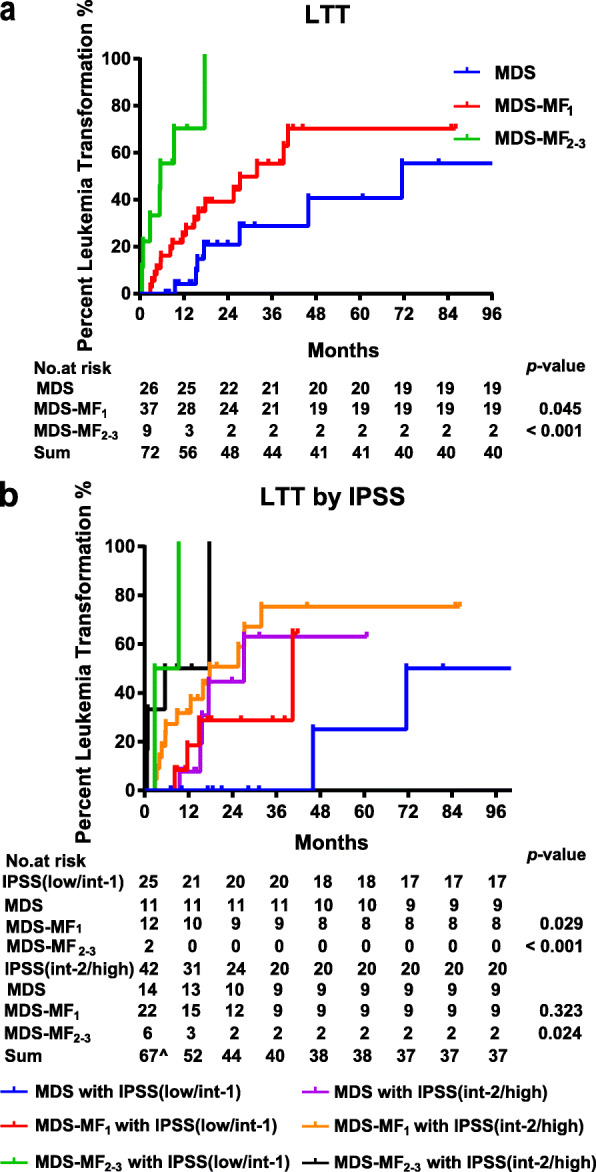
Leukemic-transformation time (LTT) curves. **A** LTT for all patients. Median LTT: 71.5 for MDS; 31.9 months for MDS-MF_1_; 5.6 months for MDS-MF_2 − 3_; MDS-MF_2 − 3_ vs. MDS *p* < 0.001; MDS-MF_1_ vs. MDS *p* = 0.045; MDS-MF_2 − 3_ vs. MDS-MF_1_*p* = 0.001. **B** LTT according to International Prognostic Scoring System (IPSS) risk categories. In the IPSS low/int-1 risk groups, median LTT: 71.5 months for MDS; 40.4 months for MDS-MF_1_; 2.8 months for MDS-MF_2 − 3_; MDS vs. MDS-MF_1_*p* = 0.029; MDS vs. MDS-MF_2 − 3_*p* < 0.001; MDS-MF_2 − 3_ vs. MDS-MF_1_*p* = 0.001. In the IPSS int-2/high risk groups, median LTT: 27.2 months for MDS; 17.8 months for MDS-MF_1_; 5.6 months for MDS-MF_2 − 3_; MDS vs. MDS-MF_1_*p* = 0.323; MDS vs. MDS-MF_2 − 3_*p* = 0.024; MDS-MF_2 − 3_ vs. MDS-MF_1_*p* = 0.131. Five Patients with IPSS int-2/high risk who refused chemotherapy; seven patients who received allogeneic-hematopoietic stem cell transplantation (4 MDS; 3 MDS-MF_1_) were excluded from LTT analysis. Two-tailed *p*-value < 0.05 by log-rank test was considered statistically significant. ^**^**^Another five patients who did not have cytogenetic analysis results (1 MDS; 3 MDS-MF_1_; 1 MDS-MF_2 − 3_) were excluded from IPSS-based LTT analysis

Cytogenetic abnormalities or genomic mutations are related to the leukemic transformation of MDS-MF but cannot account for the role of MF in this process. The leukemia clonal revolution is influenced by many factors in the bone marrow (BM) microenvironment, including inflammation and abnormal immunity. Chronic inflammation is believed to promote malignant hematopoiesis in myeloproliferative neoplasms through pro-inflammatory /fibrogenic /angiogenic cytokines [4–6]. Reactive oxygen species also play a major role in tumor progression of myeloproliferative neoplasms [7]. However, the processes responsible for the clonal myeloproliferation of MDS-MF remain unclear. It is therefore necessary to determine the variations in gene expression levels responsible for the leukemic transformation of MDS-MF. We conducted single-cell sequencing of BM mononuclear cells (BMMCs) from a patient with MDS-MF_2 − 3_ (CN) in the MDS phase (CN1) and leukemic phase (CN2). A healthy donor (NC) and a patient with *de novo* acute myeloid leukemia (AML) with the same FAB subtype (M2) were used as controls. Patients’ clinical information and detailed methods are provided in Supplementary File 1. Cell clusters were identified based on 13,280 healthy cells and BMMCs from 40 patients with newly diagnosed AML in our previous study [8] and were checked with the Human Cell Landscape (http://bis.zju.edu.cn/HCL/index.html) established by our institute [9]. Marker genes for the cell clusters are listed in Supplementary Table 2.

Seventeen cell clusters and eight cell types were identified (Fig. 2A,B). We used “blast-like” cells for the differentially expressed genes (DEGs) and gene enrichment analyses to avoid the influences of other cell types. The top 20 DEGs and their reported functions are listed in Supplementary Table 3. The top 20 increased genes during leukemic transformation included some AML-related genes, such as *CD52*, *SRGN*, *BEX1*, *BASP1*, *SPINK2*, *NEAT1*, and *CEACAM6*, and some proinflammatory mediators, such as *S100* family genes, *RNASE3*, and *CYBB*. In contrast, expression levels of many ribosomal protein genes were decreased (Fig. 2C). The *S100* family comprises proinflammatory mediators associated with acute and chronic inflammation and neoplasm metastasis [10]. *CYBB* can produce superoxide, and trigger mitochondria transfer to stimulate BM stromal cells to form AML blast cells [11]. *RNASE3* participates in nucleolysis, cell binding, lipid instability, cytotoxicity, and antibacterial activity [12]. However, none of these genes have previously been linked to leukemic transformation of MDS-MF. Gene enrichment analysis revealed that the upregulated pathways during leukemic transformation mainly contributed to inflammation /oxidation /energy metabolism-related signaling and tumor-related pathways (Fig. 2D). We also explored the DEGs between CN2 and M2 samples to detect the differences between secondary AML and *de novo* AML (Fig. 2E). Genes with higher expression in CN2 were significantly enriched in leukocyte transendothelial migration and the *Rap1* signaling pathway (Fig. 2F). Leukocyte transendothelial migration is an inflammation pathway, while *Rap1* acts as a molecular switch involved in many biological processes. *S100A12*, *RNASE3*, and *CYBB* were among the genes with higher expression levels in both CN2 compared with CN1, and in CN2 compared with M2 (Fig. 2G).

**Fig. 2:**
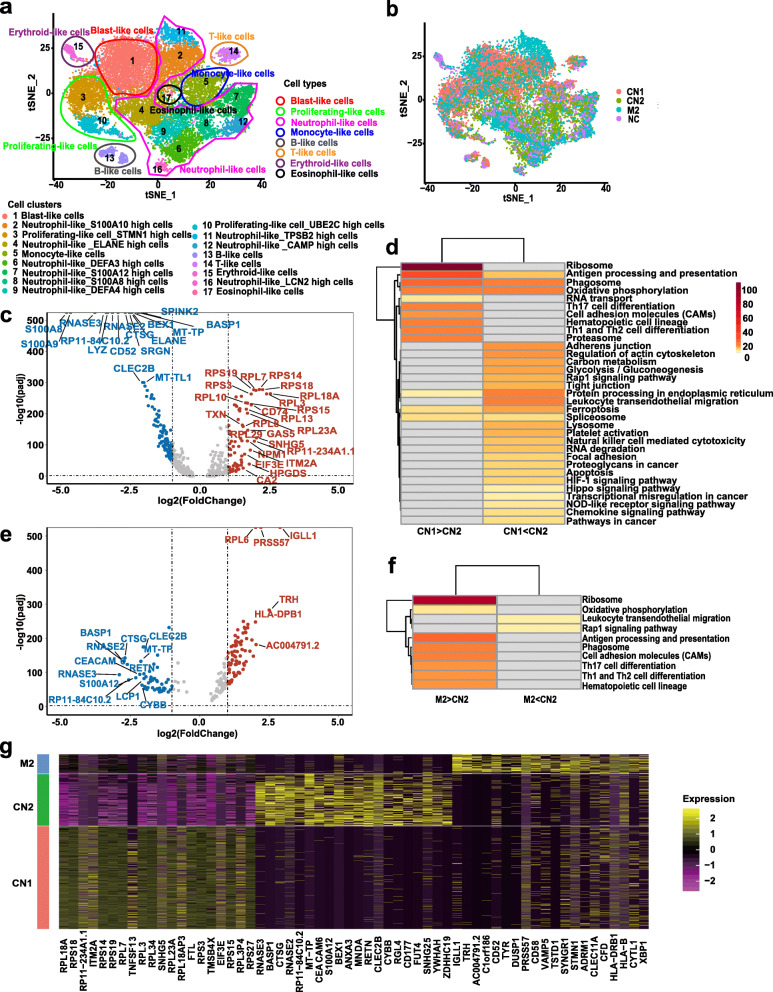
Distributions of cell clusters and single-cell gene expression patterns of blast-like clusters between CN1 and CN2, CN2 and M2. **A** Overall *t*-distributed stochastic neighbor-embedding analysis of bone marrow mononuclear cells from the four samples (NC, CN1, CN2, M2). Clusters indicated by different colors and numbers; cell types indicated by different colors of loops. **B** Overall *t*-distributed stochastic neighbor-embedding analysis of bone marrow mononuclear cells from the four samples. Samples were indicated by different colors and numbers. **C** Volcano plot of differentially expressed genes (DEGs) in blast-like cells between CN1 and CN2. The dots on the left represent higher expressed genes in CN2 and those on right represent lower in CN2. **D** Metascape Kyoto Encyclopedia of Genes and Genomes (KEGG) pathway analysis of enriched terms of DEGs in blast-like cell clusters between CN1 and CN2. Color shows *p* value. **E** Volcano plot of DEGs in blast-like cells between CN2 and M2. The dots on the left represent higher expressed genes in CN2 and those on right represent lower in CN2. **F** Metascape KEGG pathway analysis of enriched terms of DEGs in blast-like cell clusters between CN2 and M2. Color shows *p* value. **G** Heat map of DEGs among CN1, CN2, and M2 in blast-like cells. Yellow: higher expression level; red: lower expression level. Samples labeled in different colors. CN: Initial of the MDS-MF_2 − 3_ patient for single-cell sequencing; CN1: The patient CN at her MDS phase; CN2: The patient CN at her leukemic phase; NC: Normal control; M2: The patient with *de novo* AML-M_2_

In conclusion, this study revealed that MDS-MF with even mild MF had a higher risk of leukemic transformation than MDS without MF, suggesting that MDS-MF should have a different risk classification algorithm and may need special treatment. Inflammatory and oxidation activation may be essential processes, while *S100* family genes, *RNASE3*, and *CYBB* might be key genes involved in the leukemic transformation of MDS-MF.

## Supplementary information

Additional file 1 Supplementary File 1. Patients and methods.

Additional file 2 Supplementary Table 1. Characteristics of patients and treatment strategies.

Additional file 3 Supplementary Table 2. Marker genes of overall *t*-stochastic neighbor embedding map.

Additional file 4 Supplementary Table 3. Reported tumor-related functions of top-20 differentially expressed genes during leukemic transformation in the MDS-MF_2 − 3_ patient; and of top-20 differentially expressed genes between the leukemic phase of this MDS-MF_2 − 3_ patient and a *de novo* AML-M_2_ patient.

## Data Availability

The data and materials will be available upon corresponding author approval. All data sets generated/analyzed for this study are included in the manuscript and the additional files.

## Abbreviations

MDS: Myelodysplastic syndrome
MF: Myelofibrosis
MDS-MF: MDS with myelofibrosis
IPSS: International Prognostic Scoring System
Int: Intermediate
LTT: Leukemic transformation time
BM: Bone marrow
BMMC: BM mononuclear cell
AML: Acute myeloid leukemia
DEG: Differentially expressed gene
CN: MDS-MF_2 − 3_ patient for single-cell sequencing
CN1: Patient CN in MDS phase
CN2: Patient CN in leukemic phase
NC: Normal control
M2: Patient with *de novo* AML-M_2_
