# Supplementary material for: Inflammation-related genes S100s, RNASE3, and CYBB and risk of leukemic transformation in patients with myelodysplastic syndrome with myelofibrosis
Source: Biomark Res. 2021 Jul 2;9:53. doi: 10.1186/s40364-021-00304-w (PMC8259211; doi:10.1186/s40364-021-00304-w)
Supplement: Supplementary file 1 — Patients and methods. [file 40364_2021_304_MOESM1_ESM.docx]

**Patients and Methods**

This study was approved by the Institutional Review Board of the First Affiliated Hospital, Medical School of Zhejiang University, and was performed in accordance with the Declaration of Helsinki. All patients signed informed consent for participation in the research and for publication of their clinical data.

**Clinical data for retrospective patient cohort**

We retrospectively enrolled 53 patients diagnosed with *de novo* MDS-MF at our institution from 2010 to 2018. The diagnosis of MDS was made according to the 2016 WHO criteria [1]. Thirty-one control patients with *de novo* MDS without MF (MDS) were retrospectively and randomly selected during the same period. Patients with a history of MPN were excluded. Patients were followed-up until April 15, 2021, or the date of death, with a median follow-up time of 84.9 months [95% confidence interval (CI): 69.8–100 months]. Patients who were not available for follow-up for at least 6 months from diagnosis were excluded. The median follow-up times for the MDS-MF and MDS groups were 84.9 months (95% CI: 26.4–143.5 months) and 100.1 months (95% CI: 60.0–140.0 months), respectively (*p* = 0.071).

**Methods**

**MF assessment**

MF was diagnosed based on BM biopsy specimens and re-evaluated by two pathologists from our institute, and graded according to the European Consensus for BM Fibrosis Grading [2] as follows: Grade 0: absence of fibrosis, presence of scattered linear reticulin with no intersections; Grade 1: MF-1, mild fibrosis, presence of a loose network of reticulin with many intersections, especially in perivascular areas; Grade 2: MF-2, moderate fibrosis, diffuse and dense increase in reticulin with extensive intersections, occasionally with only focal bundles of collagen and/or focal osteosclerosis; and Grade 3: MF-3, severe fibrosis, diffuse and dense increase in reticulin with extensive intersections with coarse bundles of collagen, often associated with significant osteosclerosis [2]. MDS-MF was further divided into MDS with mild MF (MF grade 1, MDS-MF_1_) (n = 44) and MDS with moderate to severe MF (MF grade 2–3, MDS-MF_2-3_) (n = 9).

**Cytogenetic analysis**

BM cells were harvested directly or after 1–3 days in unstimulated culture conditions. Metaphase cells were banded by improved heat treatment and using the Giemsa R-banding method. Karyotype was determined by examining at least 20 metaphases. Two or more structural cytogenetic abnormalities, consistently additional chromosomes, or three or more metaphases for chromosome loss were required to establish abnormal clones.

**MDS-related fluorescence *in situ* hybridization (FISH)**

Five commercial probes were obtained from Vysis (Vysis Downers, Grove, IL, USA) and used to identify monomeric 7, monomeric 5, del5q31, del7q31, triploid 8, and del20q12. The cell sample was placed on a glass microscope slide and immersed in 2× SSC for 2 min at room temperature, dehydrated in graded ethanols (70%, 85%, and 100%) for 2 min, followed by adding 10 μL of probe mixture. The sample was then covered with a coverslip and sealed with rubber cement. The slides and probes were denatured at 73°C for 3 min and hybridized at 37°C overnight. After hybridization, the slides were washed and 10 μL of 4′,6-diamidino-2-phenylindole was applied. Fluorescent hybridization signals were observed using an Olympus BX60 fluorescence microscope and captured using FISH 3.0 software (Beijing GP Medical Technology Co., Ltd). Only bright and easily detectable FISH signals were used in this study. A total of 200 nuclei were evaluated for each probe. Cut-off levels for positive results were estimated at 20% of deletions and gains.

**Treatment and survival**

Patients were treated according to NCCN guidelines. Among the 84 cases, 65 (25 MDS, 34 MDS-MF_1_, 6 MDS-MF_2-3_) received chemotherapy including azacitidine or decitabine (hypomethylating agents, HMAs) alone, HMAs plus low dose cytarabine (cytarabine 10 mg/m^2^ q12h for 14 days), or low dose cytarabine alone. Twelve patients received best supportive care (BSC) including erythropoietin, granulocyte colony-stimulating factor, and transfusions. Seven patients received allogeneic hematopoietic stem cell transplantation. Five MDS-MF patients with International Prognostic Scoring System (IPSS) int-2/high risk refused chemotherapy and were treated with BSC. The above 12 patients were excluded from the survival evaluations to reduce bias. A further five patients without cytogenetic analysis results (one MDS, three MDS-MF_1_, one MDS-MF_2-3_) were excluded from IPSS-based survival analysis. There were no significant differences in the distributions of treatment strategies among the three groups (*p =* 0.520) (Supplementary Table 1). Overall survival (OS) and progression-free survival (PFS) were also analyzed by Kaplan-Meier analysis. OS and PFS were both poorer in the MDS-MF_2-3_ group (8.9 months, 95% CI: 3.1–14.7 months; 5.4 months, 95% CI: 0–13.2 months), but not in the MDS-MF_1_ group (19.1 months, 95% CI: 11.8–26.5 months; 11.2 months, 95% CI: 7.3–15.0 months), compared with the MDS group (21.0 months, 95% CI: 11.0–30.9 months; 14.3 months, 95% CI: 10.2–18.5 months) (MDS-MF_2-3_ *vs.* MDS *p* < 0.05 for OS and PFS; MDS-MF_1_ *vs.* MDS *p* > 0.05 for OS and PFS; MDS-MF_2-3_ *vs.* MDS-MF_1_ *p* < 0.05 for OS and PFS).

**Statistical analysis**

Clinical data were processed using SPSS 24 and GraphPad Prism 7 software. Survival and risk curves were prepared using the Kaplan-Meier method and log-rank test. Differences among groups were compared with nonparametric tests for continuous variables and χ^2^ tests for categorical variables. A two-tailed *p* value < 0.05 was considered significant.

**Information on patients for single-cell sequencing**

Patient 1 (MDS-MF_2-3_) (initials CN) was a 20-year-old female who was admitted to our hospital in April 2018 with a 5-day history of fever. Her white blood cell (WBC) count was 1.6 × 10^9^/L, absolute neutrophil count (ANC) was 0.2 × 10^9^/L, hemoglobin (HB) was 8.4 g/dL, and platelet count (PLT) was 70 × 10^9^/L. She had a dry tap bone marrow aspiration at her iliac bone. BM aspiration from the sternum indicated a moderate amount of nucleated cells, with 7% myeloid blast cells. Reticular-fiber staining of the iliac bone marrow biopsy indicated moderate to severe reticular-fiber hyperplasia (MF 2–3). Her karyotype showed monomeric 7, monomeric 5, del5q31, del7q31, triploid 8, and del20q12. FISH showed del5q33 95%, del5q31 95%, and triploid 8 90%. The *TP53* mutation was positive but *JAK2V617*, *JAK2E12*, *MPL*, and *CALR* mutations were all negative. She was diagnosed with MDS-EB-I with MF (MF grade=2–3), IPSS high. She received decitabine (20 mg/m^2^ for 5 days) plus ruxolitinib (15 mg bid) while waiting for allogeneic hematopoietic stem cell transplantation. She achieved hematological improvement for a while, but transformed to AML-M_2_ 4 months after diagnosis. Her BM samples at diagnosis (CN1) and at leukemic phase (CN2) were used for single-cell sequencing. Ruxolitinib was used off-label, and the patients therefore signed an informed consent for ruxolitinib treatment, which was also approved by the Ethics Review Committee of the First Affiliated Hospital of Zhejiang University School of Medicine. Because she initially had atypical MDS clinical manifestations at a very young age, including a dry tap bone marrow aspiration, blast cells with severe myelofibrosis in BM, recurrent fever without evidence of infection, weight loss, and night sweats, primary myelofibrosis or acute panmyelosis with myelofibrosis was initially considered rather than MDS; she also received an alternative treatment, and was therefore not enrolled in the retrospective study. However, she was subsequently found to have typical bone marrow morphological manifestations of dysplasia, <20% myeloid precursors, and del5q, del7q, which are MDS-specific chromosomal abnormalities, and she was finally diagnosed as MDS-EB-I with MF (MF grade=2–3), IPSS high. She also transformed to AML in a short period, and was thus selected for single-cell sequencing study.

Patient 2 (normal control, NC) was a 60-year-old female who visited our hospital in September 2017 with a 2-day history of pancytopenia. Her WBC was 3.2 × 10^9^/L, ANC was 2.5 × 10^9^/L, HB was 9.3 g /dL, and PLT was 87 × 10^9^ /L. BM aspiration was normal.

Patient 3 (*de novo* AML-M_2_, M2) was a 36-year-old female who visited our hospital in October 2017 with a 5-day history of fever. Her WBC was 13.0 × 10^9^/L, ANC 3.8 × 10^9^/L, HB 6.8 g/dL, and PLT 22 × 10^9^/L. Routine BM examination showed 22.5% blast cells, and no BM fibrosis on BM biopsy. Molecular analysis was positive for *AML1/ETO* and for *ASXL1*, *TET2*, *TP53*, and *WT1* gene mutations. Her karyotype was 45, XX, t(8,21) (q22;q22). She was diagnosed with *de novo* AML-M_2_.

**Single-cell sequencing**

BM samples obtained from an MDS-MF_2-3_ patient (CN) at diagnosis (CN1) and after transformation to AML (CN2), from a healthy donor (NC), and from a *de novo* AML patient with the same FAB subtype (M2) were used for single-cell sequencing by Microwell-sequence [3]. Cell clusters of BM mononuclear cells (BMMCs) were identified based on 13,280 healthy cells and BMMCs from 40 patients with newly diagnosed AML in our previous study [4], and were checked with the Human Cell Landscape (http://bis.zju.edu.cn/HCL/index.html) established by our institute [5].

**Cell preparation**

BMMCs were isolated using Ficoll Hypaque solution (Haoyang Institute of Biotechnology, Tianjin, China) and diluted to ~200,000/mL in Dulbecco’s Phosphate-buffered Saline (DPBS) for Microwell-sequencing. Reads were aligned to the Homo_sapiens GRCh38 genome. Only cells with >500 expressed transcripts were retained and cells with a high proportion of transcript counts derived from mitochondria-encoded genes were excluded. Gene expression was normalized and log2 transformed (TPM/100+1). The 2,000 genes exhibiting the highest cell-to-cell variation in the dataset were calculated for initial principal component analysis, and *t*-SNE analysis was then performed with the presumed number of principal components using the PCElbowPlot and JackStrawPlot functions. We then clustered the cells using the FindCluster function and applied the default Wilcoxon’s rank-sum test to detect markers differentially expressed in each cluster. Seurat3 was used to integrate different datasets for comparative analysis ([**https://satijalab.org/seurat/v3.0**](https://satijalab.org/seurat/v3.0)).

**Differentially expressed genes (DEGs)**

We analyzed DEGS in blast-like, monocyte-like, and proliferating-like cell clusters between CN1 and CN2 using the “Findmarkers” function in Seurat. Genes with a Benjamini-Hochberg-adjusted *p* value < 0.05 and absolute average log_2_ (fold change) (avg_log_2_ FC) between two groups > 0.25 were defined as DEGs. To understand the characteristics of blast-like cells from AML transformed from MDS-MF_2-3_, we also analyzed DEGs in blast-like cells between CN2 and *de novo* AML-M_2_ (M2).

**Gene enrichment analysis**

Kyoto Encyclopedia of Genes and Genomes (KEGG) enrichment analyses of DEGs was performed and presented using the online tool Metascape (http://metascape.org). KEGG pathways with a *p* value < 0.01 were considered to be signiﬁcantly enriched.

**References:**

1. Arber DA, Orazi A, Hasserjian R, Thiele J, Borowitz MJ, Le Beau MM, et al. The 2016 revision to the World Health Organization classification of myeloid neoplasms and acute leukemia. Blood. 2016;127:2391-2405.

2. Thiele J, Kvasnicka HM, Facchetti F, Franco V, van der Walt J, Orazi A. European consensus on grading bone marrow fibrosis and assessment of cellularity. Haematologica. 2005;90:1128-1132.

3. Han X, Wang R, Zhou Y, Fei L, Sun H, Lai S, et al. Mapping the mouse cell atlas by Microwell-Seq. Cell. 2018;173:1307.

4. Wu J, Xiao Y, Sun J, Sun H, Chen H, Zhu Y, et al. A single-cell survey of cellular hierarchy in acute myeloid leukemia. Journal of Hematology & Oncology. 2020;13:128.

5. Han X, Zhou Z, Fei L, Sun H, Wang R, Chen Y, et al. Construction of a human cell landscape at single-cell level. Nature. 2020;581:303-309.
