# Supplementary material for: Inflammation-related genes S100s, RNASE3, and CYBB and risk of leukemic transformation in patients with myelodysplastic syndrome with myelofibrosis
Source: Biomark Res. 2021 Jul 2;9:53. doi: 10.1186/s40364-021-00304-w (PMC8259211; doi:10.1186/s40364-021-00304-w)
Supplement: Supplementary file 2 — Supplementary Table 1. Characteristics of patients and treatment strategies. [file 40364_2021_304_MOESM2_ESM.docx]

Supplementary Table 1

**Characteristics of patients and treatment strategies**

| **Variable** | **Patients** | | **MDS** | **MDS-MF_1_** | **MDS-MF_2-3_** | | ***P* value** |
| --- | --- | --- | --- | --- | --- | --- | --- |
| **No. of patients** | 84 | | 31 | 44 | 9 | |  |
| **Age, years (median, range)** | 58(19–87) | | 58(22–87) | 56(34–85) | 59(19–71) | | 0.111 |
| **Sex M/F** | 56/28 | | 19/12 | 33/11 | 4/5 | | 0.151 |
| **Time from presenting with clinical abnormalities to receiving treatment (%)** | | | | | | | 0.623 |
| < 1 year | 66(78.6) | | 23(74.2) | 35(79.5) | 8(88.9) | |  |
| ≥ 1 year | 18(21.4) | | 8(25.8) | 9(20.5) | 1(11.1) | |  |
| **Splenomegaly (n=78) (%)^#^** | 20(25.6) | | 4/27(14.8) | 11/44 (25.0) | 3/9(33.3) | | 0.700 |
| **Constitutional symptoms (n=83) (%)^ꝉ^** | 20/83(24.1) | | 6/30(20.0) | 12/43(31.4) | 4/8(50.0) | | 0.119 |
| **Peripheral blood parameters (n=81) median (range)** | | | | | | |  |
| ANC (×10^9^/L) | 1.0(0.2–26) | | 0.80(0.2–4.9) | 1.10(0.29–26) | 0.95(0.3–3.1) | | 0.354 |
| Hemoglobin (g/L) | 68(35–143) | | 71(41–143) | 67(35–116) | 75(62–81) | | 0.335 |
| PLT (×10^9^/L) | 45(6–480) | | 45(6–331) | 52.5(8–480) | 32.0(7–89) | | 0.233 |
| **BM myeloblasts (n=83) % (range)** | 8.5(1–19) | | 7.5(1–18.5) | 9.0 (1–18.5) | 8.5(4–19) | | 0.741 |
| **World Health Organization classification (%)** | | | | | | | 0.889 |
| MDS-SLD | 9(10.7) | | 5(16.1) | 3(6.8) | 1(11.1) | |  |
| MDS-MLD | 8(9.5) | | 3(9.7) | 4(9.1) | 1(11.1) | |  |
| MDS-RS | 2(2.4) | | 1(3.2) | 1(2.3) | 0 | |  |
| MDS-EB-1 | 26(31.0) | | 8(25.8) | 14(31.8) | 4(44.4) | |  |
| MDS-EB-2 | 39(46.4) | | 14(45.2) | 22(50.0) | 3(33.3) | |  |
| MDS-U | 0 | | 0 | 0 | 0 | |  |
| MDS with isolated del(5q) | 0 | | 0 | 0 | 0 | |  |
| **Cytogenetic risk by IPSS (%) ^φ^** | 74 | | 28/31 | 39/44 | 7/9 | | 0.019**^*^** |
| Good | 37(50.0) | | 18(64.3) | 17(43.6) | 2(28.6) | |  |
| Intermediate | 20(27.0) | | 6(21.4) | 14(35.9) | 0 | |  |
| Poor | 17(23.0) | | 4(14.3) **^a^** | 8(20.5) | 5(71.4) **^a^** | | 0.006 **^*^** |
| **IPSS risk categories (%)^$^** | 79/84 | | 30/31 | 41/44 | 8/9 | | 0.561 |
| Low/intermediate-1 | 26(32.9) | | 12(40.0) | 12(29.3) | 2(25.0) | |  |
| Intermediate-2/high | 53(67.1) | | 18(60.0) | 35(70.7) | 6(75.0) | |  |
| **Treatment strategy (%)** | |  | | | | 0.520 | |
| Chemotherapy | 65(77.4) | | 25(80.6) | 34(77.3) | 6(66.7) | |  |
| HMAs alone | 28(33.3) | | 12(38.7) | 13(29.5) | 3(33.3) | |  |
| HMAs+low-dose Ara-C | 31(36.9) | | 12(38.7) | 16(36.4) | 3(33.3) | |  |
| Low-dose Ara-C alone | 6(7.1) | | 1(3.2) | 5(11.4) | 0 | |  |
| Supportive care/IST**^¶^** | 12(14.3) | | 2(6.5) **^b^** | 7(15.9)**^c^** | 3(33.3) | |  |
| Allo-HSCT | 7(8.33) | | 4(12.9) | 3(6.82) | 0 | |  |

MDS: *de novo* myelodysplastic syndrome without myelofibrosis; MDS-MF_1_: *de novo* myelodysplastic syndrome with mild myelofibrosis (MF=1); MDS-MF_2-3_: *de novo* myelodysplastic syndrome with moderate and severe myelofibrosis(MF=2-3); myelofibrosis evaluated and graded according to the European consensus for BM fibrosis grading; BM, bone marrow; MDS-SLD: MDS with single-lineage dysplasia; MDS-MLD: MDS with multilineage dysplasia; MDS-RS: MDS with ring sideroblasts; MDS-EB-1: MDS with excess blasts-1; MDS-EB-2: MDS with excess blasts-2; MDS-U: MDS, unclassifiable; IPSS: International Prognostic Scoring System; HMA: hypomethylating agent; Ara-C: cytarabine; IST: immunosuppressive therapy. Allo-HSCT: allogenic haemopoietic stem cell transplantation.

**^#^**Splenomegaly: spleen thickness measured by ultrasonography (vertical distance from splenic hilum to curvilinear tangent of opposite border of spleen) > 4.0 cm.

**^ꝉ^**Constitutional symptoms: fever ≥ 38°C, night sweat, and weight loss.

**^φ^**Chromosome banding was successfully detected in 67 cases; chromosome banding was unsuccessfully detected but FISH was performed in 7 cases.

**^$^**Defined by NCCN guidelines, version 2019.

**^¶^**Supportive care included erythropoietin (EPO) and granulocyte colony-stimulating factor (G-CSF).

**P*-value indicates comparison among MDS, MDS-MF_1_, and MDS-MF_2-3_ groups. A two-tailed P-value <0.05 was considered significant between two groups.

**^a^** Significant differences between each two groups.

**^b^** One patient with intermediate-2 risk was unwilling to receive chemotherapy.

**^c^** Of 7 patients, 1 had severe lung infection at diagnosis and was not suitable for chemotherapy; 4 refused to receive chemotherapy and were excluded from the survival evaluation, the other 2 were at low/int-1 risk.
