# Supplementary material for: Inflammation-related genes S100s, RNASE3, and CYBB and risk of leukemic transformation in patients with myelodysplastic syndrome with myelofibrosis
Source: Biomark Res. 2021 Jul 2;9:53. doi: 10.1186/s40364-021-00304-w (PMC8259211; doi:10.1186/s40364-021-00304-w)
Supplement: Supplementary file 4 — Supplementary Table 3. Reported tumor-related functions of top-20 differentially expressed genes during leukemic transformation in the MDS-MF2 − 3 patient; and of top-20 differentially expressed genes between the leukemic phase of this MDS-MF2 − 3 patient and a de novo AML-M2 patient. [file 40364_2021_304_MOESM4_ESM.docx]

**Supplementary Table 3.** Reported tumour-related functions of associated genes with expression changes during leukemic transformation in a MDS-MF patient (Gene Cards®: <https://www.genecards.org/>).

Top 20 genes with higher expression in MDS-MF patient’s leukemic phase (CN2) compared to her MDS phase (CN1) according to logFC value

| **Genes** | **avg_logFC** | **Reported functions** |
| --- | --- | --- |

Top 20 genes with higher expression in the MDS-MF patient’s leukemic phase (CN2) compared to her MDS phase (CN1), according to logFC value

| S100A8 | −4.80107 | | High expression in AML, associated with drug resistance and poor prognosis.^1^ | |
| --- | --- | --- | --- | --- |
| S100A9 | −4.65708 | | Inhibiting synthesis of erythropoietin in MDS.^2^ | |
| RNASE3 | −3.79117 | | Participating in nucleolysis, cell binding, lipid instability, cytotoxicity, and antibacterial activity.^3^ | |
| RP11-84C10.2 | −3.37971 | | lncRNAs; downregulated in AML-M_3_.^4^ | |
| LYZ | −3.34974 | | Related to medullary differentiation (also include *AZU1*, *BPI*, *CTSG*, *RNASE2*).^5^ | |
| RNASE2 | −3.15625 | | Related to medullary differentiation (also include *AZU1*, *BPI*, *CTSG*, *LYZ*).^5^ | |
| CD52 | −2.95483 | | High expression in most AML with high expression of *EVI1*.^6^ | |
| CTSG | −2.7003 | | High expression in MF; related to disorders of bone marrow stroma arrangement.^7^ | |
| SRGN | −2.55904 | | High expression in AML; poor prognosis, positive correlation with SNHG3 and Ki67.^8^ | |
| ELANE | −2.5482 | | Unfolded protein reaction encoding neutrophil elastase^9^; no literature related to AML/MDS/MF. | |
| BEX1 | −2.50829 | | Impairing cell proliferation by p53 and KRAS pathways; suggesting a good prognosis in AML with *FLT3-ITD* mutation.^10^ | |
| MT-TP | −2.46699 | | tRNA; mitochondrial disorders.^11^ | |
| BASP1 | −2.23039 | | Inhibiting proliferation of *AML1-ETO*-positive AML cells.^12^ | |
| SPINK2 | −2.18829 | | High expression in AML, central gene in occurrence and development of AML, poor prognosis.^13^ | |
| CLEC2B | −2.06673 | | Expressed on tumour cells of non-haematopoietic origins; contributing to NK cell surveillance of tumour cells.^14^ | |
| MT-TL1 | −2.01886 | | No literature related to malignant tumour. | |
| NEAT1 | −1.98204 | | *NEAT1* down-regulated in AML tissues and cells. *NEAT1* up-regulation suppressed cell growth, migration, and invasion but enhanced apoptosis of AML cells.^15^ | |
| CEACAM6 | −1.93937 | | Suggesting a poor prognosis in leukaemia.^16^ | |
| SNHG25 | −1.92841 | | lncRNA; no data available. | |
| S100A12 | −1.91319 | | Collateral homologues of S100A9 (S100A9: inhibiting synthesis of erythropoietin in MDS.^2^ | |
| Top 20 genes with higher expression in MDS-MF patient’s MDS phase (CN1) compared to her leukemic phase (CN2) according to logFC value | | | | |
| RPL18A | 2.505108 | Encoding member of L18AE family of ribosomal proteins that is component of the 60S subunit (provided by RefSeq, Jul 2012). | |  |
| RPL3 | 2.38451 | Inhibitor of cell proliferation in a p53-independent system.^17^ | |  |
| RPS18 | 2.230559 | High expression in fibroblasts and inducing keloid formation.^18^ | |  |
| RPS14 | 2.116423 | Encoding ribosomal protein that is a component of the 40S subunit (provided by RefSeq, Jul 2008). | |  |
| SNHG5 | 2.031173 | lncRNA, inhibiting progression of oesophageal cancer.^19^ | |  |
| RPL7 | 1.989143 | Intron sequence of human c-fms proto-oncogene.^20^ | |  |
| RP11-234A1.1 | 1.941038 | No literature. | |  |
| RPS19 | 1.908872 | Decreased expression leads to activation of p53 in human erythroid progenitor cells and results in failure of erythropoiesis.^21^ | |  |
| CD74 | 1.829815 | Under-expressed in AML;^22^ LGALS3 connected to CD74 in a previously unknown protein network associated with poor survival in patients with AML.^23^ | |  |
| RPS3 | 1.822382 | Only secreted by cancer cells;^24^ promoting tumorigenesis in liver cancer.^25^ | |  |
| ITM2A | 1.785854 | Arresting cell cycle in ovarian cancer.^26^ | |  |
| HPGDS | 1.759868 | Downstream of arachidonic acid/COX pathway; allergic inflammation.^27^ | |  |
| RPL23A | 1.693241 | Encoding a ribosomal protein that is a component of the 60S subunit; anticancer.^28^ | |  |
| GAS5 | 1.690796 | lncRNA, tumour suppressor across different tumour types; regulates proliferation, apoptosis, invasion, and metastasis.^29^ | |  |
| NPM1 | 1.68724 | Overexpression considered a prognostic marker of cancer recurrence and progression.^30^ | |  |
| RPS15 | 1.68625 | Inducing cell death and cell cycle arrest by p53.^31^ | |  |
| RPL13 | 1.650418 | Decreased RPL13 levels inhibit melanoma tumour development mediated through the MDM2-p53 pathway.^32^ | |  |
| RPL10 | 1.626239 | Recurrent R98S mutation in *RPL10* promotes disease progression in T-ALL.^33^ | |  |
| RPL29 | 1.612859 | High expression in colon cancer cells; inhibits cellular differentiation by p21 and p53 pathways.^34^ | |  |
| EIF3E | 1.579635 | Both oncogenic and tumour-suppressive abilities.^35^ | |  |
| Top 20 genes with higher expression in the MDS-MF patient’s leukemic phase (CN2) compared with a patient with *de novo* AML-M_2_ (M2), according to logFC value | | | |  |
| RNASE3 | −2.89751 | Participating in nucleolysis, cell binding, lipid instability, cytotoxicity, and antibacterial activity.^3^ (CN2>CN1) | |  |
| BASP1 | −2.72418 | Inhibiting proliferation of *AML1-ETO*-positive AML cells.^12^ (CN2>CN1) | |  |
| CTSG | −2.69166 | High expression in MF; related to the disorders of bone marrow stroma arrangement.^7^ (CN2>CN1) | |  |
| RNASE2 | −2.60809 | Related to medullary differentiation (also include *AZU1*, *BPI*, *CTSG*, *LYZ*).^5^ (CN2>CN1) | |  |
| RP11-84C10.2 | −2.55835 | lncRNA; downregulated in AML-M_3_.^4^ (CN2>CN1) | |  |
| S100A12 | −2.30329 | Collateral homologues of S100A9 (S100A9: inhibiting synthesis of erythropoietin in MDS.^2^) (CN2>CN1) | |  |
| CEACAM6 | −2.15976 | Suggesting a poor prognosis in leukemia.^16^ (CN2>CN1) | |  |
| MT-TP | −2.12028 | tRNA; mitochondrial disorders.^11^ (CN2>CN1) | |  |
| CYBB | −2.07820 | Producing superoxide; inhibiting apoptosis; stimulating bone marrow stromal cells to transfer mitochondria into AML cells.^36^ | |  |
| CLEC2B | −2.03287 | Expressed on tumour cells of non-haematopoietic origins; contributing to NK cell surveillance of tumour cells.^14^ (CN2>CN1) | |  |
| RETN | −2.00968 | Promoting chemotaxis of bone marrow cells;^37^ disease progression and poor survival in gliomas.^38^ | |  |
| LCP1 | −2.00514 | Related to medullary differentiation, expressing *NUP98-HOXA9* gene (associated with MPN and high-risk AML).^39^ | |  |
| CD177 | −1.97802 | Encoding neutrophil antigens; involved in human myeloproliferative disorders; a favourable prognosis in gastric cancer.^40^ | |  |
| FUT4 | −1.97665 | Promoting the malignant behaviours of leukaemia stem cells by Wnt/β-catenin pathway^41^; increasing cell proliferation by MAPK and PI3K/Akt signalling pathways.^42^ | |  |
| APLP2 | −1.93562 | Overexpressed in cancer cells; linked to increased tumour cell proliferation, migration, and invasion.^43^ | |  |
| ZDHHC19 | −1.90321 | Amplified in multiple human cancers; high levels of ZDHHC19 correlate with high levels of nuclear STAT3.^44^ | |  |
| FAM101B | −1.90133 | Family of actin regulators; regulates nuclear shape.^45^ | |  |
| ITGB2 | −1.86449 | Higher *ITGB2* expression promotes proliferation in oral squamous cell carcinoma by NADH oxidation.^46^ | |  |
| ANXA3 | −1.84254 | Correlated with drug resistance of ovarian cancer;^47^promoting development of solid tumours.^48^ | |  |
| CSTA | −1.80617 | Suppresses progression in breast cancer.^49^ | |  |
|  |  |  | |  |

Top 20 genes with higher expression in a patient with *de novo* AML-M_2_ (M2) compared with the MDS-MF patient’s leukemic phase (CN2), according to logFC value

| IGLL1 | 2.894306 | Associated with B cell-mediated immunodeficiency; high expression in gastric cancer.^50^ |
| --- | --- | --- |
| TRH | 2.501959 | High expression in AML with *TP53^mut^*.^51^ |
| PRSS57 | 2.201704 | Member of neutrophil serine protease family; promoting inflammatory response and apoptosis of CD34^+^ cells.^52^ |
| AC004791.2 | 2.02998 | lncRNA; associated with cigarette smoking.^53^ |
| RPL6 | 2.011915 | Induces drug resistance in K562/A02 cells (AML cells) by altering drug-induced apoptosis.^54^ |
| HLA-DPB1 | 2.007222 | High expression in cutaneous melanoma, immune-related gene^55^; no literature related to AML/MDS/MF. |
| C1orf186 | 1.93170 | Regulating human erythroid progenitor cell expansion and erythroblast development;^56^ predicting lymph node metastasis in endometrial endometrioid carcinoma.^57^ |
| MIF | 1.880166 | Chemokine macrophage migration inhibitory factor; induces nuclear factor-κB activation; promotes cell survival.^58^ |
| CYTL1 | 1.806775 | Inhibiting tumour metastasis with decreasing STAT3 phosphorylation in solid tumours.^59^ |
| HLA-DRB1 | 1.735023 | Antigen presentation (provided by RefSeq, Jul 2008). |
| RPL3 | 1.725391 | Inhibitor of cell proliferation in p53-independent system.^17^ (CN1>CN2) |
| NPM1 | 1.68755 | Overexpression considered prognostic marker of cancer recurrence and progression.^30^ (CN1>CN2) |
| PRSS21 | 1.669275 | Metastasis-associated ovarian cancer gene.^60^ |
| GMFG | 1.658372 | High expression correlates with poor prognosis and promotes cell migration in epithelial ovarian cancer.^61^ |
| RPL18A | 1.649859 | Encoding member of L18AE family of ribosomal proteins that is a component of the 60S subunit (provided by RefSeq, Jul 2012). (CN1>CN2) |
| SYNGR1 | 1.636536 | Encoding an integral membrane protein associated with presynaptic vesicles in neuronal cells; high expression increases ovarian cancer risk.^62^ |
| CD74 | 1.634344 | CD74 in a previously unknown protein network associated with poor survival in patients with AML.^23^ (CN1>CN2) |
| RPL10 | 1.609838 | Recurrent R98S mutation in *RPL10* promotes disease progression in T-ALL.^33^ (CN1>CN2) |
| VAMP5 | 1.605335 | Vesicle-associated membrane protein 5; correlated with poor prognosis in brain lower grade glioma.^63^ |
| EGFL7 | 1.585948 | *EGFL7* contributes to NOTCH silencing in AML resulting in shorter survival of leukemic mice.^64^ |

NC: Sample from the normal control; CN1: sample from a patient with myelodysplastic syndrome with myelofibrosis grade 2-3 (MDS-MF_2-3_) at MDS phase; CN2: sample from the same patient as CN1 at leukemic phase; M2: sample from a patient with *de novo* AML-M_2_.

**References**

1 Kovacic M, Mitrovic-Ajtic O, Beleslin-Cokic B, Djikic D, Suboticki T, Diklic M, et al. TLR4 and RAGE conversely mediate pro-inflammatory S100A8/9-mediated inhibition of proliferation-linked signaling in myeloproliferative neoplasms. *Cell Oncol (Dordr)*. 2018; **41**(5): 541– 53.

2 Giudice V, Wu Z, Kajigaya S, Fernandez Ibanez M, Rios O, Cheung F, et al. Circulating S100A8 and S100A9 protein levels in plasma of patients with acquired aplastic anemia and myelodysplastic syndromes. *Cytokine*. 2019; **113**: 462– 5.

3 Lien PC, Kuo PH, Chen CJ, Chang HH, Fang SL, Wu WS, et al. In silico prediction and in vitro characterization of multifunctional human RNase3. *Biomed Res Int*. 2013; **2013**: 170398.

4 Zimta A, Tomuleasa C, Sahnoune I, Calin G, Berindan-Neagoe I. Long Non-coding RNAs in Myeloid Malignancies. *Front Oncol*. 2019; **9**: 1048.

5 Dunne J, Cullmann C, Ritter M, Martinez Soria N, Drescher B, Debernardi S, et al. siRNA-mediated AML1/MTG8 depletion affects differentiation and proliferation-associated gene expression in t(8;21)-positive cell lines and primary AML blasts. *Oncogene*. 2006; **25**(45): 6067– 78.

6 Saito Y, Nakahata S, Yamakawa N, Kaneda K, Ichihara E, Suekane A, et al. CD52 as a molecular target for immunotherapy to treat acute myeloid leukemia with high EVI1 expression. *Leukemia*. 2011; **25**(6): 921– 31.

7 Hasselbalch H, Skov V, Stauffer Larsen T, Thomassen M, Hasselbalch Riley C, Jensen M, et al. Transcriptional profiling of whole blood identifies a unique 5-gene signature for myelofibrosis and imminent myelofibrosis transformation. *PloS one*. 2014; **9**(1): e85567.

8 Wang L, Liu H. Pathogenesis of aplastic anemia. *Hematology*. 2019; **24**(1): 559– 66.

9 Wang K, Wang M, Gannon M, Holterman A. Growth Hormone Mediates Its Protective Effect in Hepatic Apoptosis through Hnf6. *PLoS One*. 2016; **11**(12): e0167085.

10 Karasawa T, Kawashima A, Usui F, Kimura H, Shirasuna K, Inoue Y, et al. Oligomerized CARD16 promotes caspase-1 assembly and IL-1 beta processing. *Febs Open Bio*. 2015; **5**: 348– 56.

11 Auré K, Fayet G, Chicherin I, Rucheton B, Filaut S, Heckel A, et al. Homoplasmic mitochondrial tRNA mutation causing exercise-induced muscle swelling and fatigue. *Neurol Genet*. 2020; **6**(4): e480.

12 Celik H, Koh WK, Kramer AC, Ostrander EL, Mallaney C, Fisher DAC, et al. JARID2 Functions as a Tumor Suppressor in Myeloid Neoplasms by Repressing Self-Renewal in Hematopoietic Progenitor Cells. *Cancer cell*. 2018; **34**(5): 741-56.e8.

13 Xue CL, Zhang JL, Zhang GJ, Xue YY, Zhang GY, Wu X. Elevated SPINK2 gene expression is a predictor of poor prognosis in acute myeloid leukemia. *Oncol Lett*. 2019; **18**(3): 2877– 84.

14 Akatsuka A, Ito M, Yamauchi C, Ochiai A, Yamamoto K, Matsumoto N. Tumor cells of non-hematopoietic and hematopoietic origins express activation-induced C-type lectin, the ligand for killer cell lectin-like receptor F1. *Int Immunol*. 2010; **22**(9): 783– 90.

15 Feng S, Liu N, Chen X, Liu Y, An J. Long non-coding RNA NEAT1/miR-338-3p axis impedes the progression of acute myeloid leukemia via regulating CREBRF. *Cancer Cell Int*. 2020; **20**: 112.

16 Horton JD, Arbini AA, Perle MA, Raphael BG. Rapid and robust reversion to essential thrombocythemia on treatment with Decitabine in a case of hydroxyurea-induced t-MDS/AML. *Clin Case Rep*. 2016; **4**(1): 46– 50.

17 Russo A, Esposito D, Catillo M, Pietropaolo C, Crescenzi E, Russo G. Human rpL3 induces G₁/S arrest or apoptosis by modulating p21 (waf1/cip1) levels in a p53-independent manner. *Cell cycle (Georgetown, Tex)*. 2013; **12**(1): 76– 87.

18 Satish L, Lyons-Weiler, Hebda PA, Wells A. Gene expression patterns in isolated keloid fibroblasts. *Wound Repair Regen*. 2006; **14**(4): 463– 70.

19 Wei S, Sun S, Zhou X, Zhang C, Li X, Dai S, et al. SNHG5 inhibits the progression of EMT through the ubiquitin-degradation of MTA2 in oesophageal cancer. *Carcinogenesis*. 2020; 23:bgaa110.

20 Sapi E, Flick MB, Kacinski BM. The first intron of human c-fms proto-oncogene contains a processed pseudogene (RPL7P) for ribosomal protein L7. *Genomics*. 1994; **22**(3): 641– 5.

21 Pellagatti A, Hellström-Lindberg E, Giagounidis A, Perry J, Malcovati L, Della Porta M, et al. Haploinsufficiency of RPS14 in 5q- syndrome is associated with deregulation of ribosomal- and translation-related genes. *Br J Haematol*. 2008; **142**(1): 57– 64.

22 Handschuh L, Kaźmierczak M, Milewski M, Góralski M, Łuczak M, Wojtaszewska M, et al. Gene expression profiling of acute myeloid leukemia samples from adult patients with AML-M1 and -M2 through boutique microarrays, real-time PCR and droplet digital PCR. *Int J Oncol*. 2018; **52**(3): 656– 78.

23 Ruvolo P, Hu C, Qiu Y, Ruvolo V, Go R, Hubner S, et al. LGALS3 is connected to CD74 in a previously unknown protein network that is associated with poor survival in patients with AML. *EBioMedicine*. 2019; **44**: 126– 37.

24 Kim Y, Lee MS, Kim HD, Kim J. Ribosomal protein S3 (rpS3) secreted from various cancer cells is N-linked glycosylated. *Oncotarget*. 2016; **7**(49): 80350– 62.

25 Zhao L, Cao J, Hu K, Wang P, Li G, He X, et al. RNA-binding protein RPS3 contributes to hepatocarcinogenesis by post-transcriptionally up-regulating SIRT1. *Nucleic Acids Res*. 2019; **47**(4): 2011– 28.

26 Hien Nguyen TM, In-Whoan S, Lee TJ, Park J, Kim JH, Park MS, et al. Loss of ITM2A, a novel tumor suppressor of ovarian cancer through G2/M cell cycle arrest, is a poor prognostic factor of epithelial ovarian cancer. *Gynecol Oncol*. 2016; **140**(3): 545– 53.

27 Rittchen S, Heinemann A. Therapeutic Potential of Hematopoietic Prostaglandin D Synthase in Allergic Inflammation. *Cells*. 2019; **8**(6): 619.

28 Sun B, Hou Y, Hou W, Zhang S, Ding X, Su X. cDNA cloning, overexpression, purification and pharmacologic evaluation for anticancer activity of ribosomal protein L23A gene (RPL23A) from the Giant Panda. *Int J Mol Sci*. 2012; **13**(2): 2133– 47.

29 Lambrou G, Hatziagapiou K, Zaravinos A. GAS5The Non-Coding RNA and Its Role in Tumor Therapy-Induced Resistance. *Int J Mol Sci*. 2020; **21**(20).

30 Chen Y, Hu J. Nucleophosmin1 (NPM1) abnormality in hematologic malignancies, and therapeutic targeting of mutant NPM1 in acute myeloid leukemia. *Ther Adv Hematol*. 2020; **11**: 2040620719899818.

31 Daftuar L, Zhu Y, Jacq X, Prives C. Ribosomal proteins RPL37, RPS15 and RPS20 regulate the Mdm2-p53-MdmX network. *PloS one*. 2013; **8**(7): e68667.

32 Kardos G, Dai M, Robertson G. Growth inhibitory effects of large subunit ribosomal proteins in melanoma. *Pigment Cell Melanoma Res*. 2014; **27**(5): 801– 12.

33 Girardi T, Vereecke S, Sulima S, Khan Y, Fancello L, Briggs J, et al. The T-cell leukemia-associated ribosomal RPL10 R98S mutation enhances JAK-STAT signaling. *Leukemia*. 2018; **32**(3): 809– 19.

34 Liu J, Huang B, Zhang J, Carson D, Hooi S. Repression of HIP/RPL29 expression induces differentiation in colon cancer cells. *J Cell Physiol*. 2006; **207**(2): 287– 92.

35 Sesen J, Casaos J, Scotland S, Seva C, Eisinger-Mathason T, Skuli N. The Bad, the Good and eIF3e/INT6. *Front Biosci (Landmark edition)*. 2017; **22**: 1– 20.

36 Marlein CR, Zaitseva L, Piddock RE, Robinson SD, Edwards DR, Shafat MS, et al. NADPH oxidase-2 derived superoxide drives mitochondrial transfer from bone marrow stromal cells to leukemic blasts NADPH. *Blood*. 2017; **130**(14): 1649– 60.

37 Chumakov AM, Kubota T, Walter S, Koeffler HP. Identification of murine and human XCP1 genes as C/EBP-epsilon-dependent members of FIZZ/Resistin gene family. *Oncogene*. 2004; **23**(19): 3414– 25.

38 Vachher M, Arora K, Burman A, Kumar B. NAMPT, GRN, and SERPINE1 signature as predictor of disease progression and survival in gliomas. *J Cell Biochem*. 2020; **121**(4): 3010– 23.

39 Forrester AM, Grabher C, McBride ER, Boyd ER, Vigerstad MH, Edgar A, et al. NUP98-HOXA9-transgenic zebrafish develop a myeloproliferative neoplasm and provide new insight into mechanisms of myeloid leukaemogenesis. *Br J Haematol*. 2011; **155**(2): 167– 81.

40 Li Y, Mair DC, Schuller RM, Li L, Wu J. Genetic mechanism of human neutrophil antigen 2 deficiency and expression variations. *PLoS Genet*. 2015; **11**(5): e1005255.

41 Liu B, Ma H, Liu Q, Xiao Y, Pan S, Zhou H, et al. MiR-29b/Sp1/FUT4 axis modulates the malignancy of leukemia stem cells by regulating fucosylation via Wnt/β-catenin pathway in acute myeloid leukemia. *J Exp Clin Cancer Res*. 2019; **38**(1): 200.

42 Yang XS, Liu S, Liu Y-J, Liu J-W, Liu T-J, Wang X-Q, et al. Overexpression of fucosyltransferase IV promotes A431 cell proliferation through activating MAPK and PI3K/Akt signaling pathways. *J Cell Physiol*. 2010; **225**(2): 612– 9.

43 Pandey P, Sliker B, Peters H, Tuli A, Herskovitz J, Smits K, et al. Amyloid precursor protein and amyloid precursor-like protein 2 in cancer. *Oncotarget*. 2016; **7**(15): 19430– 44.

44 Niu J, Sun Y, Chen B, Zheng B, Jarugumilli G, Walker S, et al. Fatty acids and cancer-amplified ZDHHC19 promote STAT3 activation through S-palmitoylation. *Nature*. 2019; **573**(7772): 139– 43.

45 Gay O, Gilquin B, Nakamura F, Jenkins Z, McCartney R, Krakow D, et al. RefilinB (FAM101B) targets filamin A to organize perinuclear actin networks and regulates nuclear shape. *Proc Natl Acad Sci U S A*. 2011; **108**(28): 11464– 9.

46 Zhang X, Dong Y, Zhao M, Ding L, Yang X, Jing Y, et al. ITGB2-mediated metabolic switch in CAFs promotes OSCC proliferation by oxidation of NADH in mitochondrial oxidative phosphorylation system. *Theranostics*. 2020; **10**(26): 12044– 59.

47 Tong M, Fung TM, Luk ST, Ng K-Y, Lee TK, Lin C-H, et al. ANXA3/JNK Signaling Promotes Self-Renewal and Tumor Growth, and Its Blockade Provides a Therapeutic Target for Hepatocellular Carcinoma. *Stem Cell Rep*. 2015; **5**(1): 45– 59.

48 Wu N, Liu S, Guo C, Hou Z, Sun MZ. The role of annexin A3 playing in cancers. *Clin Transl Oncol*. 2013; **15**(2): 106– 10.

49 Lee S, Stewart S, Nagtegaal I, Luo J, Wu Y, Colditz G, et al. Differentially expressed genes regulating the progression of ductal carcinoma in situ to invasive breast cancer. *Cancer Res*. 2012; **72**(17): 4574– 86.

50 Hu K, Chen F. Identification of significant pathways in gastric cancer based on protein-protein interaction networks and cluster analysis. *Genetics Mol Biol*. 2012; **35**(3): 701– 8.

51 Huang R, Liao X, Li Q. TP53Identification of key pathways and genes in mutation acute myeloid leukemia: evidence from bioinformatics analysis. *Oncotargets Ther*. 2018; **11**: 163– 73.

52 Song Q, Zhao F, Yao J, Dai H, Hu L, Yu S. Protective effect of microRNA-134-3p on multiple sclerosis through inhibiting PRSS57 and promotion of CD34 cell proliferation in rats. *J Cell Biochem*. 2020; **121**(11): 4347– 63.

53 Parker M, Chase R, Lamb A, Reyes A, Saferali A, Yun J, et al. RNA sequencing identifies novel non-coding RNA and exon-specific effects associated with cigarette smoking. *BMC Med Genomics*. 2017; **10**(1): 58.

54 Chen H, Xie ZX, Jiang H, Zhang ZW, Wang GP. [Effect of ribosomal protein L6 on drug resistance and apoptosis in K562/A02 cells]. *Zhongguo Shi Yan Xue Ye Xue Za Zhi*. 2007; **15**(2): 292– 5.

55 Puig-Butille JA, Escamez MJ, Garcia-Garcia F, Tell-Marti G, Fabra A, Martinez-Santamaria L, et al. Capturing the biological impact of CDKN2A and MC1R genes as an early predisposing event in melanoma and non melanoma skin cancer. *Oncotarget*. 2014; **5**(6): 1439– 51.

56 Verma R, Su S, McCrann D, Green J, Leu K, Young P, et al. RHEX, a novel regulator of human erythroid progenitor cell expansion and erythroblast development. *J Exp Med*. 2015; **212**(6): 971.

57 Huang C, Liao K, Chou C, Shrestha S, Yang C, Chiew M, et al. Pilot Study to Establish a Novel Five-Gene Biomarker Panel for Predicting Lymph Node Metastasis in Patients With Early Stage Endometrial Cancer. *Front Oncol*. 2019; **9**: 1508.

58 Shachar I, Haran M. The secret second life of an innocent chaperone: the story of CD74 and B cell/chronic lymphocytic leukemia cell survival. *Leukemia Lymphoma*. 2011; **52**(8): 1446– 54.

59 Wang X, Li T, Cheng Y, Wang P, Yuan W, Liu Q, et al. CYTL1 inhibits tumor metastasis with decreasing STAT3 phosphorylation. *Oncoimmunology*. 2019; **8**(5): e1577126.

60 Conway G, Buzza M, Martin E, Duru N, Johnson T, Peroutka R, et al. PRSS21/testisin inhibits ovarian tumor metastasis and antagonizes proangiogenic angiopoietins ANG2 and ANGPTL4. *J Mol Med (Berlin, Germany)*. 2019; **97**(5): 691– 709.

61 Zuo P, Ma Y, Huang Y, Ye F, Wang P, Wang X, et al. High GMFG expression correlates with poor prognosis and promotes cell migration and invasion in epithelial ovarian cancer. *Gynecol Oncol*. 2014; **132**(3): 745– 51.

62 Yodsurang V, Tang Y, Takahashi Y, Tanikawa C, Kamatani Y, Takahashi A, et al. Genome-wide association study (GWAS) of ovarian cancer in Japanese predicted regulatory variants in 22q13.1. *PloS one*. 2018; **13**(12): e0209096.

63 Xiong Z, Xiong Y, Liu H, Li C, Li X. Identification of purity and prognosis-related gene signature by network analysis and survival analysis in brain lower grade glioma. *J Cell Mol Med*. 2020; **24**(19): 11607– 12.

64 Bill M, Pathmanathan A, Karunasiri M, Shen C, Burke M, Ranganathan P, et al. EGFL7 Antagonizes NOTCH Signaling and Represents a Novel Therapeutic Target in Acute Myeloid Leukemia. *Clin Cancer Res*. 2020; **26**(3): 669– 78.
